# Experience of Treating *Candida auris* Cases at a General Hospital in Qatar

**DOI:** 10.1017/ash.2021.119

**Published:** 2021-07-29

**Authors:** Adila Shaukat, Feah Visan, Naser Al Ansari, Walid Al Wali, Manal Hamed, Ihab Elmadhoun, Hassan Mitwally, Edin Karic

## Abstract

**Background:** So far, there have been no studies on *Candida auris* in Qatar. We describe the clinical spectrum and outcome of *C. auris* infection in patients admitted to a general hospital in Qatar. **Methods:** We conducted this descriptive observational study in a general hospital in Qatar. We have included all patients with *C. auris* infection and colonization admitted to a general hospital from December 2018 to August 2019. **Results:** We identified 13 patients with confirmed *C. auris* infection or colonization, of whom 5 cases represented an actual *C. auris* infection, while the remaining 8 cases were considered colonization. The mean age of the patients with infection was 76.6 years (SD, ±8.4), while the mean age of the patients with colonization was 66.4 years (SD, ±24.7). Among the individuals clinically infected with *C. auris*, 2 had urinary tract infections, 1 had candidemia, 1 acquired a soft-tissue infection, and 1 had a lower respiratory tract infection. All strains of *C. auris* were susceptible to echinocandins, flucytosine, and posaconazole while resistant to fluconazole and amphotericin B. Of the patients with *C. auris* infection who received systemic antifungal therapy, 3 (60%) died during antifungal therapy. **Conclusions:** Our study showed that *C. auris* can cause a wide variety of invasive infections, including bloodstream infection, urinary tract infection, skin infection, and lower respiratory tract infections, especially in critically ill patients. In addition, our isolates showed resistance to the most common antifungal agents such as fluconazole and amphotericin B.

**Funding:** No

**Disclosures:** None

